# Clinical utility and reliability of reducing blood culture incubation to 4 days in pediatric patients

**DOI:** 10.1128/jcm.01125-26

**Published:** 2026-10-12

**Authors:** Saina Beitari, Jennifer Dien Bard

**Affiliations:** 1 Department of Pathology and Laboratory Medicine, Children’s Hospital Los Angeles, Los Angeles, California, USA; 2 Keck School of Medicine, University of Southern California 5116, Los Angeles, California, USA; Maine Medical Center Department of Medicine, Portland, Maine, USA

**Keywords:** pediatrics, incubation period, blood culture

## LETTER

Blood culture is the mainstay for diagnosing bloodstream infections, providing critical diagnostic results that may reduce morbidity and mortality in pediatric sepsis (1, 2). While standard protocols rely on a 5-day incubation period, emerging data suggest a 4-day protocol preserves diagnostic sensitivity while optimizing laboratory efficiency (3–5). However, most studies focused on adult populations, and the efficacy in children is less well established. We retrospectively evaluated a 4-day incubation standard at our quaternary care pediatric center.

**Fig 1:**
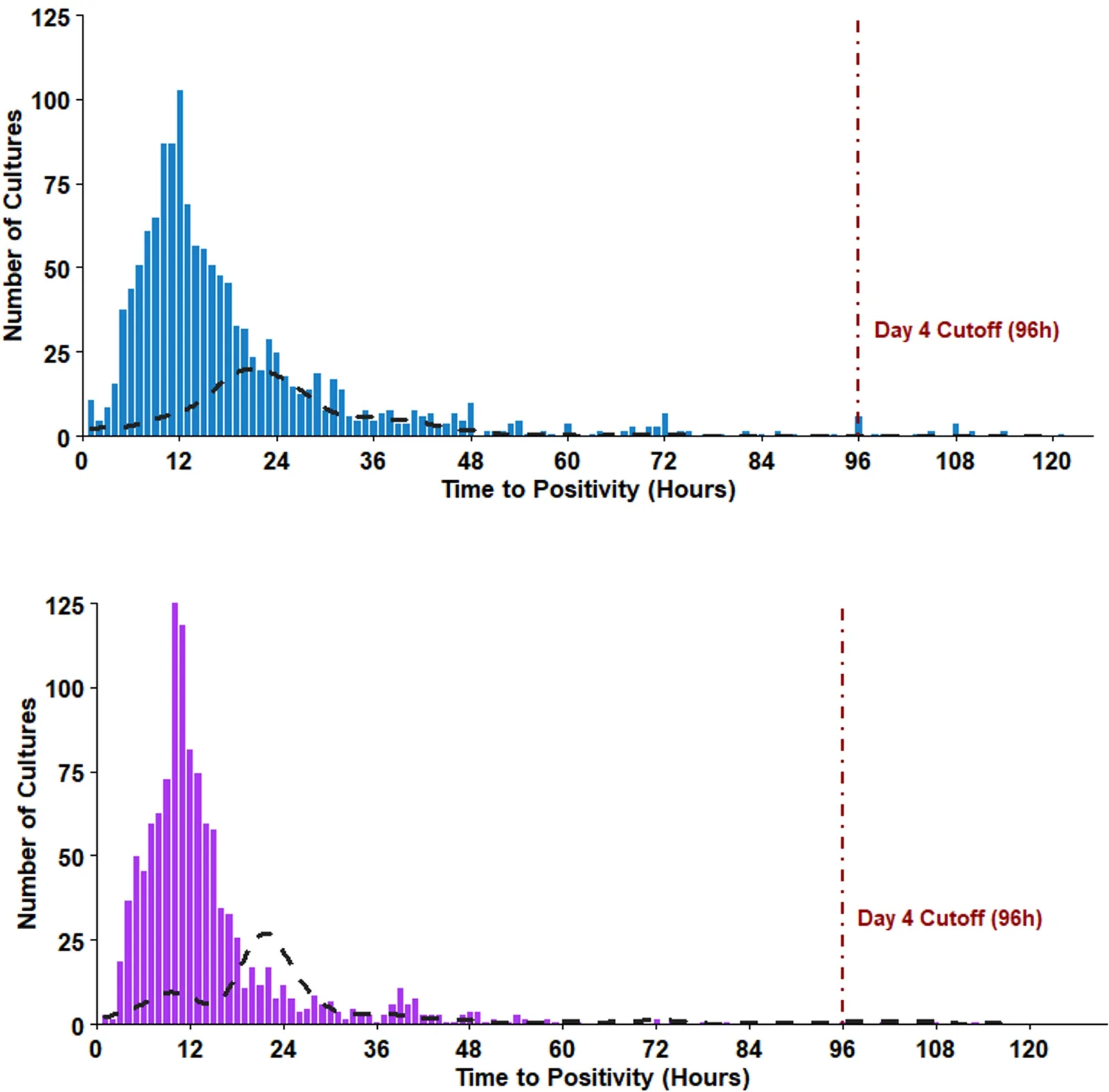
Distribution of positive blood culture bottles by time to positivity (TTP) classified by true pathogen (bars) and contaminants (dashed-line curve). The dashed vertical line indicates Day 4 cutoff (96 h). Top panel, aerobic bottles. Bottom panel, anaerobic bottles.

Between January 2020 and January 2025, we analyzed 55,825 blood culture bottles (26,352 aerobic, 29,473 lytic anaerobic; BD BACTEC FX, Sparks, MD, USA) from 14,804 patients (median age, 3 years; interquartile range [IQR], 10 years). The overall positivity rate was 6.1%, with a higher positivity rate from aerobic bottles (6.7% [1,779/26,352]) than from anaerobic bottles (5.5% [1,627/29,473]; *P* < 0.0001) (Table S1). Time-to-positivity (TTP) analysis showed 99.0% of aerobic and 98.7% of anaerobic blood culture bottles flagged positive within 4 days. Potential contaminants, as defined by the College of American Pathologists (6, 7), had a significantly longer TTP than true pathogens regardless of blood culture bottle types (Fig. 1). Median TTP for contaminants versus pathogens was 22.5 h (IQR, 17.8–29.1) versus 14.0 h (IQR, 10.0–23.0) in aerobic bottles, and 22.0 h (IQR, 18.0–27.9) versus 11.5 h (IQR, 9.0–16.0) in anaerobic bottles. A marked temporal decline was observed in blood cultures yielding true pathogens, with 83.0% positive within Day 1, 12.7% within Day 2, 2.6% within Day 3, and 1.0% within Day 4. Overall, diagnostic yield declined significantly after 4 days, yielding only 33 (0.06%) positives (15 aerobic, 18 anaerobic; Table S1). Notably, all fungal organisms recovered from blood cultures were detected prior to the 4-day cutoff with a median TTP of 15.0 h (IQR, 10–23 h).

The 33 delayed positive blood cultures from 29 patients were assessed for clinical significance, defined as antimicrobial modifications or central line removal in response to the positive blood (Table 1), and 32 (97.0%) of the cases from 28 patients were considered insignificant or were known positives. Specifically, 17 blood cultures from 17 (51.5%) patients were considered contaminants requiring no intervention, including *Cutibacterium acnes* (median TTP, 108 h) recovered from 13 (39.4%) cases. The remaining 16 (48.5%) blood cultures from 12 patients grew potential pathogens after the 4-day threshold. Among the 12 patients, 11 (92.9%) were known positives with prior blood cultures recovering the same organism within 4 days, including four blood bottles positive for *Mycobacterium* species. While a single delayed culture grew *Arcanobacterium haemolyticum,* the organism was previously recovered from a concurrent nares drainage, and the patient was already on directed therapy.

**TABLE 1:** Diagnostic yield and clinical impact of delayed blood culture bottles

Organism	Blood culture bottle type	No. of bottles	Considered true pathogen	Median time to positivity (h)	Prior blood culture positive within 4 days	Positive blood culture after 4 days drove change in management	Clinical action taken
*Arcanobacterium haemolyticum*	Anaerobic	1	Yes	108	No (prior positive culture from nare drainage)	No	Maintained on active therapy
*Bifidobacterium breve*	Aerobic	1	Yes	99	Yes	No	Maintained on active therapy
Coagulase-negative *Staphylococcus*	Aerobic	1	No	116	No	No	None; considered contaminant
*Corynebacterium amolycolatum*	Aerobic	1	Yes	121	No	No	None; considered contaminant
*Cutibacterium acnes*	Anaerobic	13	No	108	Yes (2/13) No (11/13)	No (13/13)	None; considered contaminant (13/13)
*Escherichia coli*	Aerobic	1	Yes	108	Yes	No	Maintained on active therapy
*Fusobacterium nucleatum*	Anaerobic	1	Yes	113	Yes	No	Maintained on active therapy
Gram-positive (non-viable)	Anaerobic	1	Yes	101	No	No	None; considered contaminant
*Haemophilus parainfluenzae*	Aerobic	3	Yes	105	Yes (3/3)	No (3/3)	Maintained on active therapy (3/3)
*Klebsiella oxytoca*	Aerobic	1	Yes	108	Yes	No	Maintained on active therapy
*Klebsiella pneumoniae*	Aerobic	1	Yes	110	Yes	No	Maintained on active therapy
*Mycobacterium mucogenicum*	Aerobic	4	Yes	109	Yes (4/4)	No (4/4)	Maintained on active therapy (4/4)
*Mycobacterium immunogenum*	Aerobic	1	Yes	108	Yes	No	Maintained on active therapy
*Pseudomonas aeruginosa*	Aerobic	1	Yes	98	Yes	No	Maintained on active therapy
*Staphylococcus epidermidis*	Anaerobic	1	Yes	100	Yes	No	Maintained on active therapy
*Staphylococcus hominis*	Anaerobic	1	No	106	Yes	No	None; considered contaminant

As treatment duration for certain bloodstream infections such as catheter-related infections may depend on documentation of microbiologic clearance (8), we additionally reviewed whether delayed positive blood culture affected the documented date of bloodstream clearance or duration of antimicrobial therapy (Table S2). Overall, none of the 11 patients with delayed positive blood cultures (*n* = 15) resulted in extension of antimicrobial duration or line removal. Specifically, in six patients, the organism was detected in a same-day concurrent blood culture. Three patients had delayed blood cultures collected 1 day after the initial positive, including one with *Haemophilus parainfluenzae* endocarditis already receiving a planned 4-week course of intravenous ceftriaxone. Despite a delayed positive blood culture collected 3 days after the initial positive in a patient with *Klebsiella oxytoca* bacteremia, line removal was prompted by persistent bacteremia confirmed by a subsequent peripheral culture that flagged within 17 h. In the remaining patient with *Mycobacterium immunogenum* bacteremia, a delayed positive collected 10 days after the initial positive did not alter therapy. This study does have limitations, which include the absence of blood culture bottle fill volume data, uncaptured pre-analytical transit times from patient collection to incubator loading, and the inclusion of occasional unpaired blood culture bottles.

In conclusion, of the 3,406 positive aerobic and anaerobic blood culture bottles encountered, 0.03 (*n* = 1) of true pathogens were not recovered within 4 days, justifying a shortened 4-day incubation standard in pediatrics. In fact, 95.7% of all positives occur within 2 days of incubation. This operational shift may optimize laboratory workflows and maintain diagnostic sensitivity, without adversely affecting clinical management.

## SUPPLEMENTAL MATERIAL

Supplemental tables jcm.01125-26-s0001.docx Tables S1 and S2.

ASM does not own the copyrights to Supplemental Material that may be linked to, or accessed through, an article. The authors have granted ASM a non-exclusive, world-wide license to publish the Supplemental Material files. Please contact the corresponding author directly for reuse.
