# Supplementary material for: Clinical utility and reliability of reducing blood culture incubation to 4 days in pediatric patients
Source: J Clin Microbiol. 2026 Oct 12;64(11):e01125-26. doi: 10.1128/jcm.01125-26 (PMC13753834; doi:10.1128/jcm.01125-26)
Supplement: Supplementary file 1 [file jcm.01125-26-s0001.docx]

**Table S1. Aerobic and anaerobic blood culture bottle positivity rates, stratified by day of detection.**

|  | **Aerobic** | | **Anaerobic** | | **Total** | |
| --- | --- | --- | --- | --- | --- | --- |
| **Time to positivity** | **True Pathogen,**  **N (%)** | **Contaminant,**  **N (%)** | **True Pathogen,**  **N (%)** | **Contaminant,**  **N (%)** | **True Pathogen,**  **N (%)** | **Contaminant,**  **N (%)** |
| **Day 1 (0-24h)** | 1066 (78.2%) | 244 (58.6%) | 1062 (88.4%) | 285 (66.9%) | 2128 (83.0%) | 529 (62.8%) |
| **Day 2 (24-48h)** | 215 (15.8%) | 156 (37.5%) | 110 (9.2%) | 97 (22.8%) | 325 (12.7%) | 253 (30.0%) |
| **Day 3 (48-72h)** | 46 (3.4%) | 13 (3.1%) | 21 (1.7%) | 16 (3.7%) | 67 (2.6%) | 29 (3.4%) |
| **Day 4 (72-96h)** | 22 (1.60%) | 2 (0.5%) | 5 (0.4%) | 13 (3.0%) | 27(1.0%) | 15 (1.8%) |
| **Day 5 (96-120h)** | 14 (1%) | 1 (0.3%) | 3 (0.2%) | 15 (3.5%) | 17 (0.6%) | 16 (1.9%) |
| **Total** | 1363 | 416 | 1201 | 426 | 2564 | 842 |

Abbreviation: N, number

**Table S2: Summary of patients with delayed blood culture positive after initial blood culture positivity within 4-day incubation**

| **Patient** | **Organism recovered** | **Collection source** | **Initial blood culture bottle type** | **Delayed blood culture bottle (number)** | **Time to positivity of delayed bottle**  **(days)** | **Difference between initial and delayed culture collection (days)** | **Delayed positive collected within same day as initial (Yes/No)*** | **Central line removal prompted by delayed positive** |
| --- | --- | --- | --- | --- | --- | --- | --- | --- |
| 1 | *Bifidobacterium breve* | Venous-peripheral stick | Anaerobic | Aerobic (1) | 4.13 | 0 | Yes | No |
| 2 | *Escherichia coli* | PICC | Aerobic | Aerobic (1) | 4.50 | 0 | Yes | No^#^ |
| 3 | *Fusobacterium nucleatum* | CVC | Anaerobic | Anaerobic (1) | 4.71 | 0 | Yes | No |
| 4 | *Haemophilus parainfluenzae* | Venous-peripheral stick | Aerobic | Aerobic  (3) | 4.38  4.77  4.38 | 0  1  1 | Yes | N/A |
| 5 | *Klebsiella oxytoca* | PICC | Aerobic | Aerobic (1) | 4.5 | 3^ | No | No^^^ |
| 6 | *Klebsiella pneumoniae* | CVC | Aerobic | Aerobic (1) | 4.58 | 1 | No | No^#^ |
| 7 | *Mycobacterium mucogenicum* | CVC | Aerobic | Aerobic  (3) | 4.75  4.31  4.5 | 0  1  1 | Yes | No^#^ |
| 8 | *Mycobacterium mucogenicum* | CVC | Aerobic | Aerobic (1) | 4.54 | 1 | No | No^#^ |
| 9 | *Mycobacterium immunogenum* | CVC | Aerobic | Aerobic (1) | 4.5 | 10~ | No | No^#^ |
| 10 | *Pseudomonas aeruginosa* | CVC | Aerobic | Aerobic (1) | 4.08 | 1 | No | No^#^ |
| 11 | *Staphylococcus epidermidis* | CVC | Aerobic | Anaerobic (1) | 4.17 | 0 | Yes | No |

*Abbreviations:* CVC: Central Venous Catheter; PICC: Peripherally Inserted Central Catheter; N/A: Not Applicable.

* Second positive blood culture was either part of same blood culture set or a different draw within a 24-hour period.

^#^ Line removal decision was driven by the initial positive blood culture result.

^ An additional blood culture via venous-peripheral stick was positive within 17 hours of collected. Line removal was prompted by the additional positives.

~Between the initial positive blood culture and the delayed blood culture collected 10 days later, there were 8 blood culture bottles collected, and all were negative. Initial antimicrobial regimen continued with no extension.
